# Safety of sublingual immunotherapy for asthma with standardized house dust mite vaccines in a tropical setting: Results of a nation-wide pharmacovigilance study

**DOI:** 10.1186/1939-4551-8-S1-A40

**Published:** 2015-04-08

**Authors:** Maytee Mateo Morejón, Alexis Labrada, Raúl Lázaro Castro Almarales, Giset Jiménez López, Ismary Alfonso Orta, Mary Carmen Reyes Zamora

**Affiliations:** 1 National Center of Bioproducts, Cuba; 2 Center for State Control of Drugs, Medical Devices (CEDMED), Cuba

## Background

Pharmacovigilance studies are helpful to follow the use of allergen immunotherapy in clinical practice. Industrially manufactured allergen vaccines (VALERGEN) of three mite species (including the tropical species Dermatophagoides siboney and Blomia tropicalis) have been recently introduced country-wide in Cuba for asthma treatment. To assess the safety and efficacy of allergen immunotherapy by injection or sublingual routes, using VALERGEN vaccines in the routine clinical practice.

## Methods

A national prospective study was conducted using the cohort adverse event monitoring method. Patients have been followed at least for 12 months. The study has included 2108 asthmatic patients above 5 years old, attending 24 allergy services in 10 provinces. Efficacy variables were measured using a standard questionnaire, reporting data on the last two weeks prior to the administration.

## Results

52.5% of included patients received SLIT and 47.5% SCIT, 51.3% were children. Overall, 101 adverse reactions were reported. The frequency of local reactions per administration was 0.16% and 0.013%, for SCIT and SLIT, respectively; whereas the frequency of systemic reactions was significantly greater for SCIT (0.204%) versus SLIT (0.003%). All systemic reactions by SLIT were classified as mild whereas 2 severe (Grade IV) reactions were reported by SCIT. The frequency of both local and systemic reactions was significantly greater in adults as compared to children. On the other hand, significant increase of Quality of Life index, decrease of asthma severity and frequency of asthma manifestations and drug consumption was found, as compared to pretreatment values for both administration routes in a similar extent.

## Conclusions

The results confirm the efficacy of SCIT and SLIT using standardized house dust mite allergen vaccines for asthma treatment, as well as the higher safety profile of the sublingual route.

